# Low and Decreasing Prevalence and Rate of False Positive HIV Diagnosis — Chókwè District, Mozambique, 2014–2017

**DOI:** 10.15585/mmwr.mm6749a3

**Published:** 2018-12-14

**Authors:** Daniel Shodell, Robert Nelson, Duncan MacKellar, Ricardo Thompson, Isabelle Casavant, Didier Mugabe, Sherri Pals, Dawud Ujamaa, Juvencio Bonzela, Judite Cardoso, Salvador Machava, Carlos Lourenço, Chunfu Yang, Bharat Parekh, Ishani Pathmanathan, Andrew F. Auld, Stelio Tamele, Manuel Antonio Ouane, Vania Macome, Noela Chicuecue, Guita Amane, Luciana Kohatsu, Nely Honwana, Stanley Wei, Peter R. Kerndt, Edgar Monterroso, Alfredo Vergara

**Affiliations:** ^1^CDC Country Office, Maputo, Mozambique; ^2^Division of Global HIV and TB, Center for Global Health, CDC; ^3^Chókwè Health Research and Training Center, National Institute of Health, Chókwè, Mozambique; ^4^Jhpiego, Johns Hopkins University, Maputo, Mozambique; ^5^Chókwè District Public Health Directorate, Chókwè, Mozambique; ^6^FHI 360, Maputo, Mozambique; ^7^Mozambique Ministry of Health, Maputo, Mozambique.

## Abstract

In 2017, rapid human immunodeficiency virus (HIV) testing services enabled the HIV diagnosis and treatment of approximately 15.3 million persons with HIV infection in sub-Saharan Africa with life-saving antiretroviral therapy (ART) (*1*). Although suboptimal testing practices and misdiagnoses have been reported in sub-Saharan Africa and elsewhere, trends in population burden and rate of false positive HIV diagnosis (false diagnosis) have not been reported (*2*,*3*). Understanding the population prevalence and trends of false diagnosis is fundamental for guiding rapid HIV testing policies and practices. To help address this need, CDC analyzed data from 57,655 residents aged 15–59 years in the Chókwè Health and Demographic Surveillance System (CHDSS) in Mozambique to evaluate trends in the rate (the percentage of false diagnoses among retested persons reporting a prior HIV diagnosis) and population prevalence of false diagnosis. From 2014 to 2017, the observed rate of false diagnosis in CHDSS decreased from 0.66% to 0.00% (p<0.001), and the estimated population prevalence of false diagnosis decreased from 0.08% to 0.01% (p = 0.0016). Although the prevalence and rate of false diagnosis are low and have decreased significantly in CHDSS, observed false diagnoses underscore the importance of routine HIV retesting before ART initiation and implementation of comprehensive rapid HIV test quality management systems (*2*,*4*,*5*).

Located in Gaza Province of southern Mozambique, CHDSS conducts annual demographic surveillance of approximately 100,000 residents of Chókwè District. In 2017, an estimated 25.6% of residents aged 15–59 years had HIV infection (*6*). During 2014–2017, staff members visited all CHDSS households in each of four surveillance rounds and offered a brief survey and HIV testing to household members aged 15–59 years. In the first surveillance round (April 2014–April 2015), all consenting participants who reported a prior HIV diagnosis were tested in accordance with the national rapid HIV test algorithm (NRTA). In subsequent surveillance rounds, consenting participants who reported a prior HIV diagnosis were offered, but not required, to test for HIV infection. Dried blood spots from participants with NRTA-negative or indeterminate results who reported a prior diagnosis of HIV infection were tested at CDC with a serologic testing algorithm followed by ultrasensitive HIV-1 gp41 total nucleic acid polymerase chain reaction, if negative by serology (Figure) (*7*). Before delivering CDC-confirmed HIV-negative test results, participants were reinterviewed to verify their prior HIV diagnosis and were retested a second time in accordance with the NRTA. Participants who confirmed their prior diagnosis and retested HIV-negative were informed that they had been misdiagnosed, provided counseling and psychosocial support, and disengaged from HIV care in coordination with their HIV care provider.

**FIGURE:**
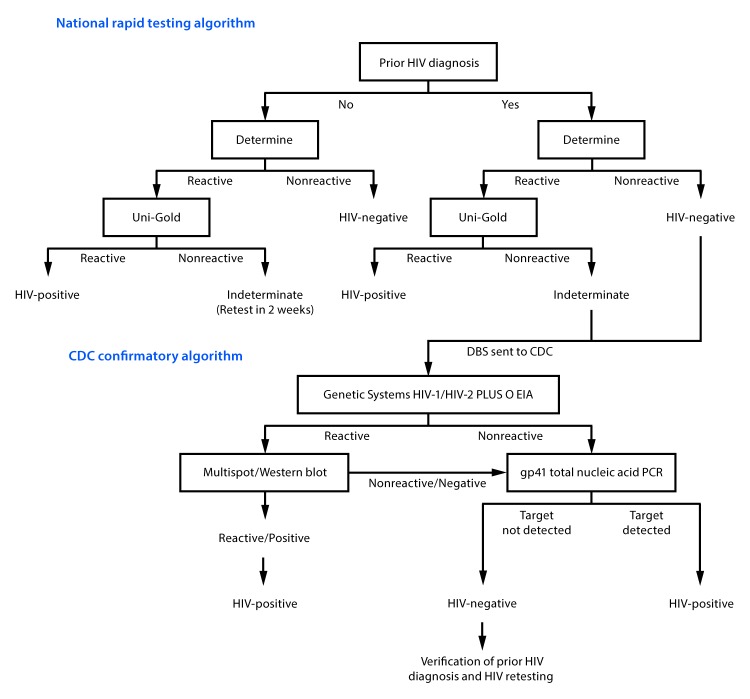
National rapid* and CDC confirmatory HIV testing algorithms for survey participants aged 15–59 years who reported having received a prior HIV diagnosis — Chókwè Health Demographic Surveillance System (CHDSS), Chókwè, Mozambique, 2014–2017 **Abbreviations:** DBS = dried blood spot; EIA = enzyme immunoassay; gp41 = glycoprotein 41; HIV = human immunodeficiency virus; PCR = polymerase chain reaction. * The Mozambique national rapid test algorithm refers to the use of Determine followed by Uni-Gold in accordance with national HIV testing guidelines. Prior HIV diagnosis is defined as reporting during the CHDSS survey of 1) ever having tested HIV-positive, 2) testing HIV-positive at the last test, or 3) currently or ever receiving HIV care.

To estimate the prevalence of false diagnosis in the second and subsequent surveillance rounds, cases were imputed by applying the observed cumulative false diagnosis rate to nontested participants who reported a prior HIV diagnosis. Logistic regression was used to test for linear trends in the observed rate and estimated prevalence of false diagnosis across surveillance rounds, adjusting for within-household correlation. Maximum expected cases, rates, and prevalence of false diagnosis were calculated using the World Health Organization (WHO) prequalification lower 95% confidence limits for sensitivity and specificity for Determine* and Uni-Gold^†^ rapid HIV tests (*8*,*9*). Excess cases were calculated as the difference between total estimated and maximum expected false diagnoses.

During 2014–2017, among 57,655 CHDSS residents aged 15–59 years, 43,496 (75.4%) participated in at least one round of surveillance (Table 1). Prior HIV diagnosis, based on the Mozambique national HIV testing algorithm (Figure), was reported by 8,608 (19.8%) participants, among whom 5,568 (64.7%) were tested for HIV. Of those tested, >99.0% in all demographic groups were NRTA-positive, including 4,698 (99.6%) of 4,719 participants who reported being on ART.

**TABLE 1:** National rapid and CDC confirmatory HIV test outcomes among survey participants aged 15–59 years who reported having received a prior HIV diagnosis, by selected characteristics and round of participation — Chókwè Health Demographic Surveillance System (CHDSS), Mozambique, 2014–2017

Characteristic	CHDSS residents and survey participants			National rapid HIV testing algorithm			CDC confirmatory testing		
	No. of residents*	Survey participants^†^ no. (%)	Prior HIV diagnosis^§ ^no. (%)	HIV tested^¶^ no. (%)	HIV-positive no. (%)	HIV-negative/indet,** no. (%)	DBS tested^††^ no. (%)	HIV-positive^§§ ^no. (%)	HIV-negative^¶¶^ no. (%)
**All survey rounds**									
**Total**	**57,655**	**43,496 (75.4)**	**8,608 (19.8)**	**5,568 (64.7)**	**5,534 (99.4)**	**34 (0.6)**	**33 (97.1)**	**3 (9.1)**	**30 (90.9)**
**Sex**									
Female	35,378	28,339 (80.1)	6,755 (23.8)	4,471 (66.2)	4,444 (99.4)	27 (0.6)	26 (96.3)	1 (3.8)	25 (96.2)
Male	22,277	15,157 (68.0)	1,853 (12.2)	1,097 (59.2)	1,090 (99.4)	7 (0.6)	7 (100.0)	2 (28.6)	5 (71.4)
**Age group (yrs)**									
15–24	26,306	19,886 (75.6)	1,073 (5.4)	614 (57.2)	609 (99.2)	5 (0.8)	5 (100.0)	0 (—)	5 (100.0)
25–34	13,482	9,678 (71.8)	2,637 (27.2)	1,639 (62.2)	1,634 (99.7)	5 (0.3)	5 (100.0)	0 (—)	5 (100.0)
35–59	17,867	13,932 (78.0)	4,898 (35.2)	3,315 (67.7)	3,291 (99.3)	24 (0.7)	23 (95.8)	3 (13.0)	20 (87.0)
Survey round***									
1 (2014–2015)	51,362	24,947 (48.6)	3,169 (12.7)	3,169 (100.0)	3,145 (99.2)	24 (0.8)	23 (95.8)	3 (13.0)	20 (87.0)
2 (2015–2016)	47,823	24,455 (51.1)	2,623 (10.7)	1,232 (47.0)	1,226 (99.5)	6 (0.5)	6 (100.0)	0 (—)	6 (100.0)
3 (2016–2017)	47,624	24,178 (50.8)	1,865 (7.7)	805 (43.2)	801 (99.5)	4 (0.5)	4 (100.0)	0 (—)	4 (100.0)
4 (2017)	48,556	20,302 (41.8)	951 (4.7)	362 (38.1)	362 (100.0)	0 (—)	0 (—)	0 (—)	0 (—)

**Abbreviations:** DBS = dried blood spots; HIV = human immunodeficiency virus; indet = indeterminate.

* Approximately 102,500 persons of all ages were residents in CHDSS during round 1. For each survey round, counselors visited each household in CHDSS (20,122 households in round 1) and offered all available household members aged 15–59 years the opportunity to participate in a brief survey and to test for HIV.

^†^ Totals for sex and age groups include residents who participated in any one of four survey rounds. For these characteristics, counts are unique individuals. Survey rounds 2, 3, and 4 include some residents who participated in a prior survey round. Within each round, counts reflect unique individuals. The sum of rounds include repeat participants.

^§^ Reporting during the survey of ever having tested HIV-positive, testing HIV-positive at the last test, or currently or ever receiving HIV care. Percentages are of survey participants. Participants who reported a prior HIV diagnosis in more than one round are counted only once in the round in which they first reported receiving a prior HIV diagnosis. Including repeat participants, 4,778 (20%), 5,440 (22%), and 4,539 (22%) residents reported a prior HIV diagnosis in survey rounds 2, 3, and 4, respectively. Excludes 12 participants who on follow-up were verified not to have received a prior HIV diagnosis.

**^¶^** In round 1, counselors collected a 1-mL whole blood specimen from all consenting participants who reported a prior HIV diagnosis. Specimens were tested for HIV at the CHDSS research laboratory by trained laboratory technicians in accordance with the national rapid test algorithm. In rounds 2–4, consenting participants who reported a prior HIV diagnosis were encouraged but not required to test for HIV if they had not previously tested HIV-positive as part of CHDSS. Participants who reported a prior diagnosis and who consented to test were HIV tested at home by trained counselors in accordance with the national rapid HIV test algorithm. Percentages are of participants who reported a prior HIV diagnosis.

** Four participants tested HIV-indeterminate.

^††^ Excludes 12 participants who on follow-up were verified not to have received a prior HIV diagnosis. Dried blood spots of participants who reported a prior HIV diagnosis and who tested HIV-negative or indeterminate by the national rapid test algorithm were shipped on dry ice and tested at CDC in accordance with a standard confirmatory testing algorithm.

^§§^ All had tested HIV-indeterminate by the national rapid testing algorithm.

**^¶¶^** Of 27 (90%) persons contacted at follow-up, 27 (100%) retested HIV-negative in accordance with the national rapid HIV test algorithm a median of 170 days (interquartile range = 142–263 days) from their survey encounter.

*** Round 1: April 2014–April 2015; round 2: May 2015–January 2016; round 3: March 2016–March 2017; round 4: April 2017–November 2017.

CDC confirmatory testing was conducted on specimens from 45 of 46 NRTA-negative or indeterminate participants who initially reported a prior HIV diagnosis. All 41 NRTA-negative participants tested HIV-negative at CDC; three of four NRTA-indeterminate participants tested HIV-positive, and one tested HIV-negative. Of 42 CDC-confirmed HIV-negative participants, 39 were recontacted, and 12 (31%) verified that they had never tested HIV-positive. Reasons for initial misclassification included interviewer error, or participant misunderstanding, perceived need to report a diagnosis to receive services, or mental illness. Among the 27 recontacted participants who confirmed their prior HIV diagnosis, all retested NRTA-negative a median of 170 days (interquartile range = 142–263 days) after their survey encounter. Overall, 31 participants were classified as having received a false diagnosis, including one participant who had insufficient specimen for confirmatory testing and three CDC-confirmed HIV-negative participants lost to follow-up (Table 2).

**TABLE 2:** Number of observed, estimated, and maximum expected false positive HIV diagnosis (false diagnosis) cases, and rates and prevalence of false diagnosis among survey participants aged 15–59 years, by selected characteristics and surveillance round — Chókwè Health Demographic Surveillance System (CHDSS), Mozambique, 2014–2017

Characteristic	False diagnosis rate*		False diagnosis prevalence		Maximum expected false diagnosis outcomes^†^			
	No. of observed cases	False diagnosis rate^§^ % (95% CI)	Total estimated no. of cases^¶^	False diagnosis prevalence** % (95% CI)	No. of expected cases^††^	False diagnosis rate^§§ ^%	False diagnosis prevalence^¶¶ ^%	No. of excess cases***
**Total**	**31**	**0.56 (0.36–0.75)**	**48**	**0.11 (0.08–0.13)**	**4**	**0.047**	**0.009**	**44**
**Sex**								
Female	26	0.58 (0.36–0.80)	39	0.13 (0.10–0.17)	3	0.041	0.011	36
Male	5	0.46 (0.06–0.85)	9	0.07 (0.04–0.09)	1	0.058	0.006	8
**Age group (yrs)**								
15–24	5	0.81 (0.10–1.53)	9	0.04 (0.02–0.06)	2	0.179	0.010	7
25–34	5	0.31 (0.04–0.57)	9	0.10 (0.05–0.14)	1	0.029	0.010	8
35–59	21	0.63 (0.36–0.90)	31	0.22 (0.15–0.28)	1	0.024	0.007	30
**Survey round^†††^**								
1 (2014–2015)	21	0.66 (0.38–0.94)^§§§^	21	0.08 (0.04–0.12)^¶¶¶^	1	0.047	0.004	20
2 (2015–2016)	6	0.49 (0.10–0.87)^§§§^	14	0.05 (0.02–0.08)^¶¶¶^	1	0.047	0.004	13
3 (2016–2017)	4	0.50 (0.01–0.98)^§§§^	10	0.04 (0.02–0.07)^¶¶¶^	1	0.047	0.004	9
4 (2017)	0	0.00 (0.00–0.01)^§§§^	3	0.01 (0.00–0.03)^¶¶¶^	0	0.047	0.000	3

**Abbreviations:** CI = confidence interval; HIV = human immunodeficiency virus.

* Includes 27 persons who reported a prior HIV-positive diagnosis and tested HIV-negative in accordance with the national rapid test and CDC confirmatory test algorithms, and at follow-up a median of 170 days (interquartile range = 142–263 days) from their survey encounter, retested HIV-negative in accordance with the national rapid HIV test algorithm, and reaffirmed that they had received a prior HIV-positive diagnosis. Also includes four participants who reported a prior HIV-positive diagnosis and tested HIV-negative in accordance with the national rapid test algorithm, but who had insufficient specimen for testing at CDC (one participant), or were confirmed HIV-negative at CDC but were lost to follow-up for retesting and confirmation of reported prior HIV diagnosis (three participants). Excludes 12 participants who on follow-up were verified not to have received a prior HIV diagnosis.

^†^ Maximum outcomes were calculated using standard formulae and reported lower 95% confidence limits (LCL) for sensitivity (SENS) and specificity (SPEC) from World Health Organization prequalification studies: Alere Determine HIV-1/2 (D): WHO report PQDx 0033–013–00 (https://www.who.int/diagnostics_laboratory/evaluations/pq-list/hiv-rdts/160712_amended_final_public_report_0033_013_00_v5.pdf); LCL for sensitivity and specificity for Determine are 99.10 (SENS_DLCL_) and 97.80 (SPEC_DLCL_), respectively. Uni-Gold HIV (U): WHO report PQDx 0149–052–00 (https://www.who.int/diagnostics_laboratory/evaluations/pq-list/hiv-rdts/171103_amended_final_pqpr_0149_052_00_v7.pdf); LCL for sensitivity and specificity for Uni-Gold are 98.70 (SENS_ULCL_) and 99.20 (SPEC_ULCL_), respectively.

^§^ 1 – positive predictive value of self-reported prior HIV-positive diagnosis (PPV_SRDx_); PPV_SRDx_ = (No. prior HIV diagnoses – No. observed false diagnoses)/No. prior HIV diagnoses.

^¶^ Includes 17 false diagnosis cases imputed for rounds 2–4, calculated by applying the overall and demographic subgroup-specific false diagnosis rates to nontested survey participants who reported having received a prior HIV-positive diagnosis. Sum of estimated cases by age group (49) does not equal total estimated cases (48) because of rounding error.

** Total false diagnoses/survey participants, weighted to CHDSS census age-group, sex, and urban/rural distribution.

^††^ Prior HIV diagnosis x Maximum expected false diagnosis rate, rounded up to the nearest integer. Sum of expected cases by survey round (3) does not equal total expected cases (4) because of rounding error.

^§§^ 1 – lowest expected positive predictive value (PPV) of national rapid test algorithm (PPV_NRTA-LE_). PPV_NRTA-LE_ = (PREV*SENS_NRTA-LE_) / [(PREV*SENS_NRTA-LE_) + (1-PREV)(1- SPEC_DLCL_)(1-SPEC_ULCL_)]; SENS_NRTA-LE_ = SENS_DLCL_*SENS_ULCL_. PREV = observed round 1 HIV prevalence: total, 27.8%; female, 30.3%; male, 23.6%; aged 15–24 years, 9.1%; aged 25–34 years, 38.5%; aged 35–59 years, 43.1%; rounds 1–4, 27.8%.

^¶¶^ Maximum expected false diagnoses/survey participants, weighted to CHDSS census age-group, sex, and geographic distribution.

*** Difference between total estimated and maximum expected false diagnosis cases. Sum of estimated cases by age group (45) and survey round (45) does not equal total estimated cases (44) because of rounding error.

^†††^ Round 1: April 2014–April 2015; round 2: May 2015–January 2016; round 3: March 2016–March 2017; round 4: April 2017–November 2017.

^§§§^ Test for linear trend: p<0.001. Round 4 one-sided 97.5% upper confidence limit is estimated using Clopper-Pearson method. https://jamanetwork.com/journals/jama/fullarticle/385438.

^¶¶¶^ Test for linear trend: p = 0.0016.

During 2014–2017, the observed rate of false diagnosis in CHDSS decreased from 0.66% to 0.00% (p<0.001), and estimated prevalence of false diagnosis decreased from 0.08% to 0.01% (p = 0.0016) (Table 2). The cumulative observed false diagnosis rate and estimated prevalence of false diagnosis were 0.56% and 0.11%, respectively. Compared with maximum expectations based on WHO prequalification studies, 44 excess false diagnoses were estimated overall, decreasing from 20 in the first round (2014–2015) to three in the fourth round (2017) (Table 2).

## Discussion

False positive HIV diagnosis can result in severe individual and public health consequences, including separation from spouse and family, unnecessary care and treatment, and public distrust in HIV testing. Accurate estimation of the population burden and trends in false diagnosis is therefore critical for guiding rapid HIV testing policies and practices. In a high HIV prevalence district in Mozambique, among 5,568 residents who reported a prior HIV diagnosis, including 4,719 on ART, nearly all (>99.0%) tested HIV-positive with the Mozambique NRTA. Both the low observed rate (0.66%) and estimated prevalence (0.08%) of false diagnosis in the first round of surveillance (2014–2015) decreased to nearly zero by the fourth round (2017). Nonetheless, applying the estimated cumulative false diagnosis prevalence of 0.11% to the estimated 100,421 residents aged 15–64 years in Chókwè District, 110 residents might have ever received a false diagnosis.

As with all diagnostic tests that have excellent, but not perfect performance, false positive HIV diagnoses are expected even when testing is conducted in accordance with standard procedures and with approved, multitest algorithms (*2*,*3*). Compared with WHO prequalification expectations, 20 excess false diagnoses were observed in the first round of surveillance, decreasing to an estimated three cases in the fourth round. Although reasons for excess false diagnoses are unclear, findings from the CHDSS are consistent with reports suggesting that the specificity of the Determine rapid HIV test can be lower than manufacturer claims (*2*,*3*,*10*). Observed reductions in excess false diagnoses might be attributed to improved rapid HIV test practices and quality management systems or increased client-initiated retesting among persons who are diagnosed (*4*). Provider-initiated retesting before ART initiation as recommended by WHO was not routinely implemented during this period (2014–2017) and most likely does not account for observed reductions in false HIV diagnoses (*2*).

Notably, the observed cumulative rate of false positive HIV diagnosis in the CHDSS (0.56%) is less than one fifth the median false diagnosis rate (3.1%) reported in a recent systematic review of 30 studies (*3*). Findings of the low cumulative and decreasing rate of false diagnosis in the CHDSS are reassuring, and caution should be exercised in interpreting results of this systematic review. Higher rates of false diagnosis reported in many studies might be attributed to the use of suboptimal testing strategies such as a third rapid test as a tiebreaker to rule in HIV infection and lack of verification of HIV diagnostic claims (*3*). Lack of verification might be a particularly important limitation, as nearly one third of reinterviewed CHDSS participants who were initially classified as having had a false positive HIV diagnosis were verified to have never received an HIV diagnosis. Studies that do not include follow-up procedures to verify self-reported HIV diagnoses might substantially overreport false diagnosis.

After being informed of their misdiagnosis, nearly all contacted participants expressed relief that they were not infected and no longer needed HIV care. At the request of one participant, the psychologist and medical officer from the local health authority confirmed the client’s status with concerned family members; no other follow-up support services were requested. All contacted participants were successfully disengaged from HIV care, including 16 who were on ART. Public concerns about the accuracy of HIV testing and reductions in uptake of rapid HIV testing services in Chókwè District have not been reported.

The findings in this report are subject to at least four limitations. First, after the first round, fewer than half of participants who claimed a prior diagnosis were tested for HIV. Estimated cases and prevalence of false diagnosis, however, is conservative because imputed cases were based on the higher cumulative false diagnosis rate rather than lower round-specific rates, and participants who did not complete all testing and prior-diagnosis verification steps were assumed to have received false diagnoses. Second, surveillance of quality management system activities among facility and community rapid HIV test providers was not conducted, and the potential impact of these activities on reducing the rate and prevalence of false diagnosis is unknown. Third, it is possible that some HIV-infected participants who were receiving ART might have false negative test results because of loss of detectable antibody (*2*,*3*,*7*). Total nucleic acid polymerase chain reaction is not 100% sensitive, and retesting negative does not rule out HIV infection for patients on ART (*7*). Participants who discontinued ART are being retested periodically. Finally, this study was conducted in a high HIV prevalence district in southern Mozambique. Because the positive predictive value of diagnostic tests depends, in part, on disease prevalence, other areas and districts of Mozambique might have higher rates of false diagnosis attributed to lower HIV prevalence alone.

Low and decreasing trends in the estimated prevalence of false positive HIV diagnosis in CHDSS indicate that residents in Chókwè District have received high-quality rapid HIV testing services, and that HIV care and ART is provided near universally to only those in need. However, observed false diagnoses in Chókwè District underscore the importance of routine retesting and confirmation of HIV infection for all patients before ART initiation, and implementation of comprehensive quality management systems to ensure appropriate training, supervision, proficiency testing, and external quality assessment of rapid HIV test providers (*2*,*4*,*5*).

**Summary**

**What is already known about this topic?**

A systematic review of studies conducted in sub-Saharan Africa suggests higher than expected rates of false positive human immunodeficiency virus (HIV) diagnosis (false diagnosis) using rapid tests.

**What is added by this report?**

From 2014 to 2017, the rate and population prevalence of false diagnosis in Chókwè District, Mozambique, decreased from 0.66% to 0.00% and from 0.08% to 0.01%, respectively. The cumulative false diagnosis rate was 0.56%, less than one fifth the median rate (3.1%) reported in the systematic review.

**What are the implications for public health practice?**

Low and decreasing prevalence and rate of false diagnosis are reassuring and underscore caution in extrapolating results of the systematic review. Nonetheless, observed false diagnoses underscore the need for routine HIV retesting before initiation of antiretroviral therapy and implementation of comprehensive rapid HIV test quality management systems.

## Footnotes

*
https://www.who.int/diagnostics_laboratory/documents/guidance/determine.pdf .

† https://www.who.int/diagnostics_laboratory/documents/guidance/uni_gold.pdf.
